# Management of soft tissues in patients with periprosthetic joint infection

**DOI:** 10.1186/s42836-023-00205-3

**Published:** 2023-10-03

**Authors:** Hongyi Shao, Yixin Zhou

**Affiliations:** Department of Orthopaedic Surgery, Beijing Jishuitan Hospital, Capital Medical University, Fourth Clinical College of Peking University, No. 31 Xinjiekou East Street, Xicheng District, Beijing, 100035 China

**Keywords:** Periprosthetic joint infection, Soft tissues, Management

## Abstract

**Background:**

Appropriate soft tissue management represents a critical step in treating periprosthetic joint infection (PJI). This review discusses relevant guidelines that surgeons should follow in the management of soft tissues in PJI treatment.

**Body:**

It is imperative for arthroplasty surgeons to thoroughly debride and rebuild soft tissue with a good blood supply. Relevant guidelines that surgeons should follow rigorously include preoperative evaluation of soft tissue status and plan-making, adequate surgical area exposure, intraoperative removal of all necrotic and infected soft tissues, adequate coverage of soft tissue defects, timely postoperative assessment and management of soft tissues, wound management and proper rehabilitation.

**Conclusion:**

Soft tissue management plays a critical role in the treatment of PJI. To improve the infection control rate and postoperative joint function, surgeons should be familiar with these general principles and rigorously practice them in PJI management.

## Background

Management of soft tissue is a critical step in the treatment of periprosthetic joint infection (PJI) [1]. Appropriate soft tissue management provides a better local blood supply, rendering it easier to control infection [2, 3]. However, the soft tissue per se may be involved in PJI. Currently, it is imperative for arthroplasty surgeons to thoroughly debride and reconstruct soft tissues with a good blood supply [4, 5]. In the handling of PJI, the surgeon should follow the relevant guidelines rigorously, such as careful evaluation of preoperative status of soft tissues, intraoperative removal of all necrotic and infected soft tissues, adequate coverage of soft tissue defects, and the timely postoperative assessment and management of soft tissues.

## Preoperative evaluation and planning

**Fig. 1:**
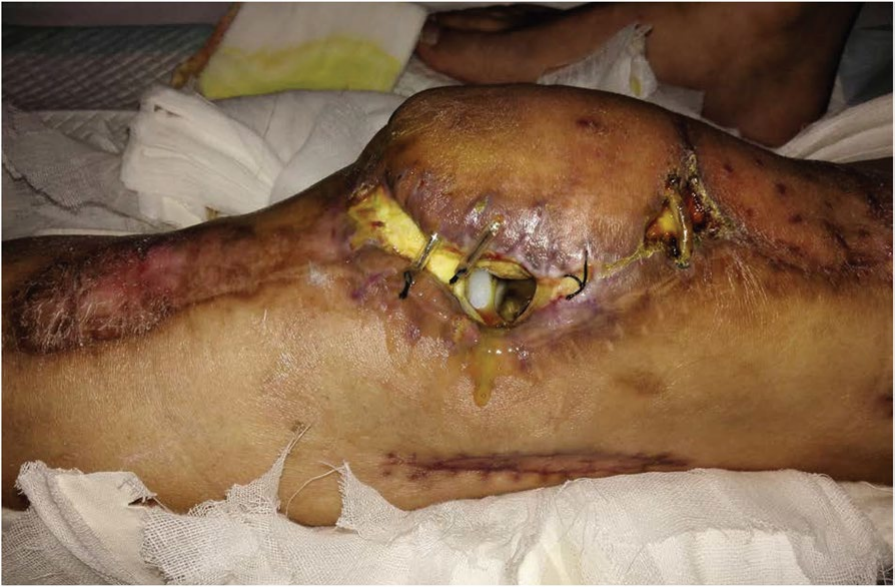
Soft tissue defects after total knee arthroplasty

Clinically, PJI may have different manifestations, such as sinus tracts, severe swelling and infection of local soft tissues, the loss of range of motion (ROM) leading to soft tissue fibrosis, and even soft tissue defects (Fig. 1). However, a problem shared by all postoperative patients is a surgical scar that surgeons must take this into consideration.

Whenever we plan to make the incision on the prior incision line, it should be incorporated into the revised surgical plan. If the prior surgical incision site is not suitable, the proposed surgical approach/incision should go perpendicular to it, thus preserving as much blood supply to soft tissues as possible. In other words, the angle formed by both prior and currently planned incisions should be as close to 90 degrees as possible [6]. If the presently proposed surgical incision runs parallel to the previous one, the two should be at least 5–7 cm apart [7]. For the knee joint, if multiple surgical incisions are made, the lateral incision should be chosen because the blood supply to the soft tissues of anterior knee principally comes from the medial side [6].

Both the preoperative extent of soft tissue infection and soft tissue quality are robust predictors of treatment outcomes [8]. Although magnetic resonance imaging (MRI) is not included in the PJI diagnostic criteria formulated by the Musculoskeletal Infection Society [9], it is still valuable in some cases, especially in those who are likely to develop recurrent infections or have undetected abscesses [10]. MRI coupled with metal artifact reduction protocols can help in identifying periprosthetic bone and soft tissue abnormalities, including intramuscular edema, subcutaneous fluid collection, and the extent of infection and these findings can inform surgical planning [11].

**Fig. 2:**
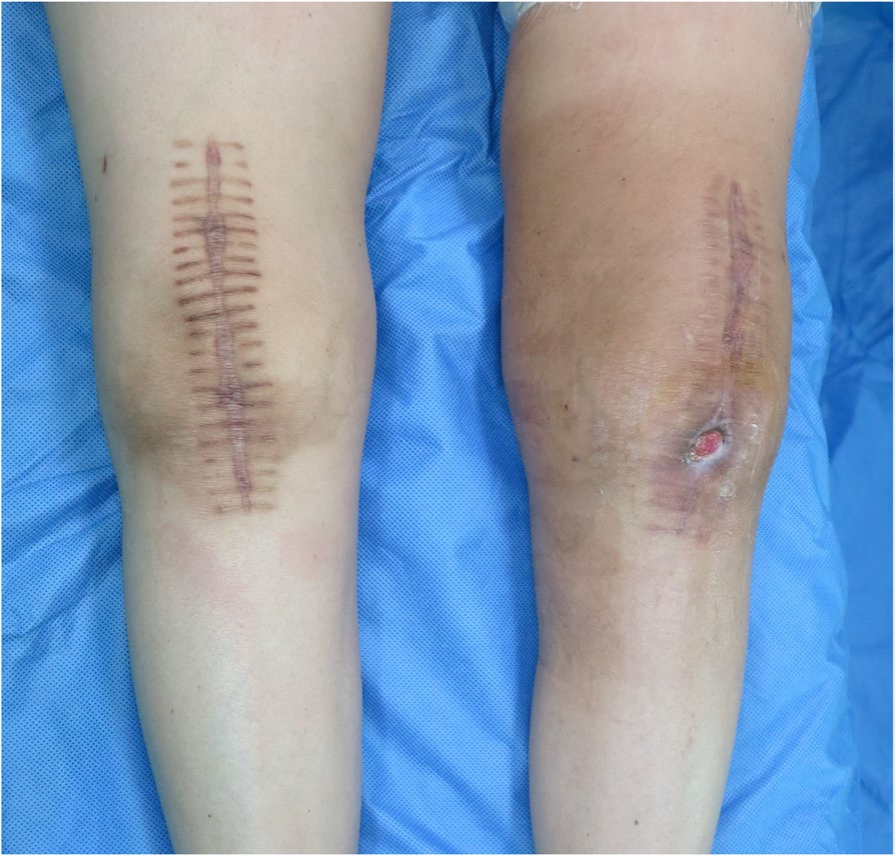
The patient developed left knee PJI after bilateral total knee arthroplasty. The sinus is situated just above the tibial tubercle

Skin defects or sinus tracts that develop after joint surgery pose a great challenge for orthopedic surgeons (Fig. 2). If a sinus is located at the site of a surgical incision, direct resection can be considered. If the sinus lies relatively far away from the surgical incision, circumferential resection can be an option, but it is worth mentioning that the two incisions should be at least 2.5–5 cm apart [12]. Guidelines about skin flap repair after joint surgery are not currently available. If there is a relatively large skin defect after joint surgery or if the defect becomes too large after sinus surgery, the assistance of a plastic surgeon should be sought immediately.

## Intraoperative management of soft tissues

### Exposure to the surgical area

**Fig. 3:**
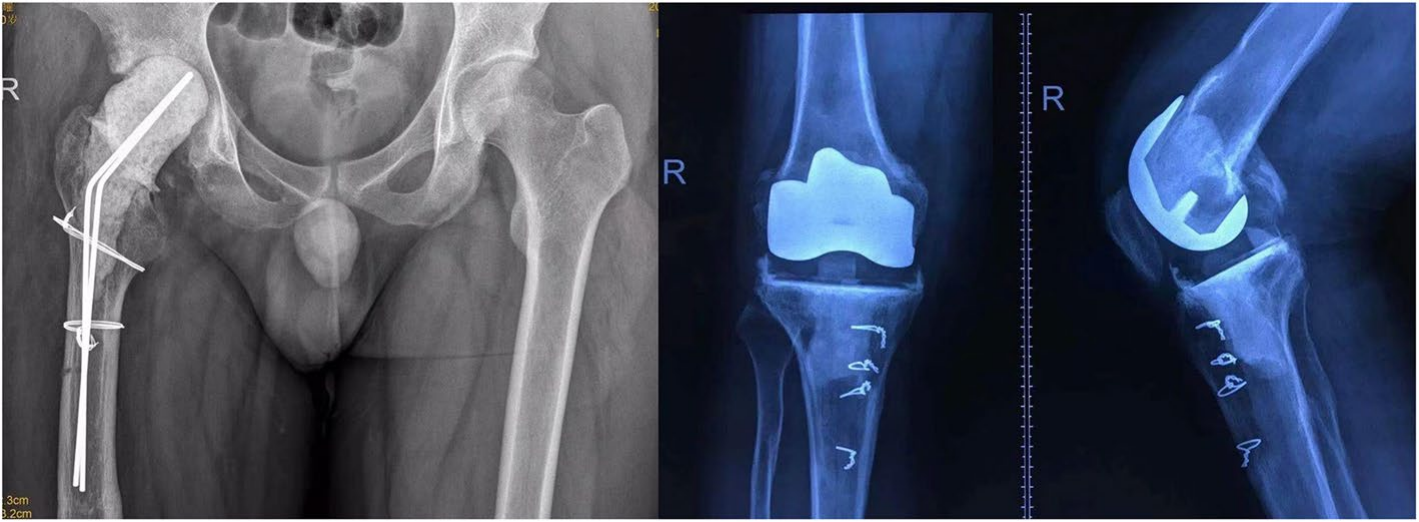
**A** Extended trochanteric osteotomy in a hip revision patient and (**B**) tibial tubercle osteotomy in a knee revision case

Thorough debridement is critical for successful treatment of PJI [13]. Achieving adequate exposure to surgical areas is an essential step of debridement [14]. A previous operation or multiple prior operations may cause scar hyperplasia around the joint, especially in patients with loss of ROM [15]. For these patients, a substantial/sufficient soft tissue release is necessary to attain good exposure. Surgeons need to remove the scar around the joint meticulously. Extended trochanteric osteotomy is required in some hip revision patients and tibial tubercle osteotomy is needed in some knee revision cases to accomplish sufficient exposure (Fig. 3).

**Fig. 4:**
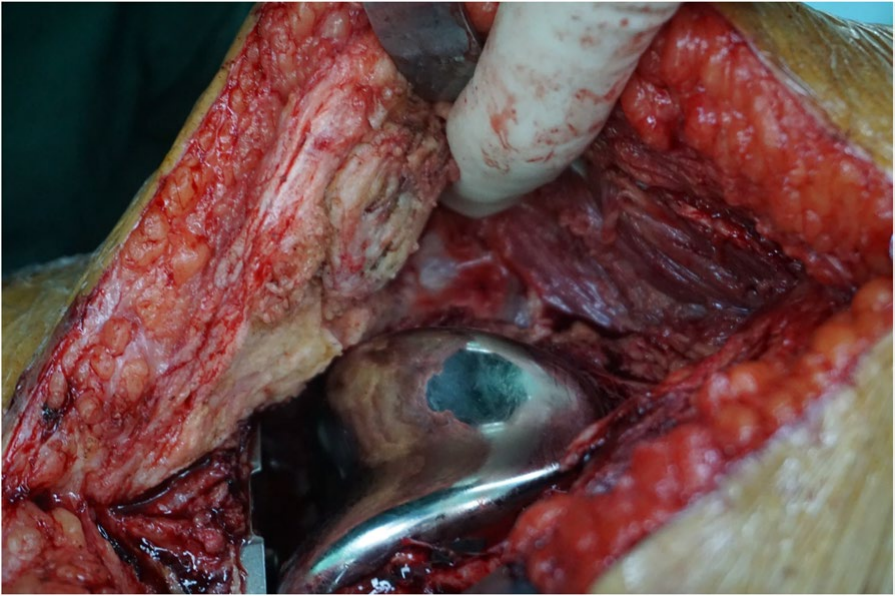
Lateral gutter release after knee arthrotomy

**Fig. 5:**
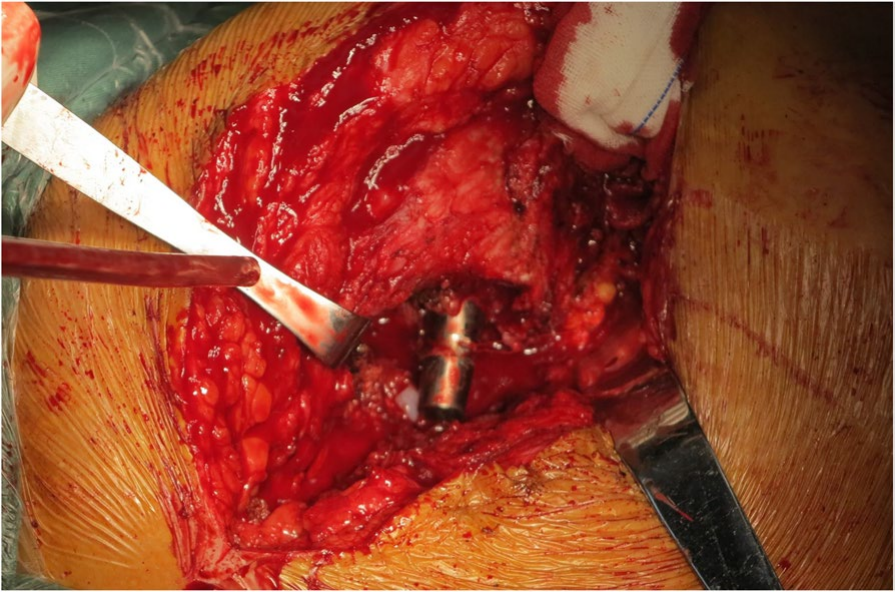
Release of the anterior superior acetabulum to expose the acetabulum

Once the joint is opened, scar formation caused by a previous surgery necessitates extensive release and removal of the scar tissues. For knee, the medial and lateral gutter should be released so that the surgeon can flex it (Fig. 4). With hips, scar tissues around the hip joint should be removed so that the surgeon can mobilize the femur to expose the acetabulum (Fig. 5).

### Intraoperative removal of infected soft tissues

As aforementioned, some soft tissues in the joints of PJI patients can become infected. Hence, these necrotic or infected tissues should be completely removed during debridement. Thus, orthopedic surgeons should make every effort to remove infected or necrotic tissues as much as possible while preserving normal and healthy tissues [16]. The critical issue, however, lies in distinguishing between necrotic or infected tissues and healthy ones. Orthopedic surgeons should consider the following factors to avoid soft tissue defects whenever possible: (1) whether the ligaments or fascia is intact, which can prevent the spread of infection; (2) that muscles should have sufficient blood supply; and (3) that no soft tissues, such as the synovium, are short of blood supply.

### Dealing with soft tissue defects

Soft tissue defects are a challenging problem with debridement in PJI patients. After explantation of the prothesis, a static spacer may be used if there were ligament or muscle defects [17]. However, for fascia or skin defects, repair with skin flaps may sometimes be necessary, especially in knee patients. The most commonly used skin flaps are medial gastrocnemius rotational flaps. Other skin flaps include free flaps, lateral gastrocnemius flaps, quadriceps advancement flaps, local fascio-cutaneous flaps, etc. [18]. Usually, a plastic surgeon’s assistance is required in these situations. The International Consensus Meeting has concluded that when a prosthesis is explanted, reconstructive surgery using skin flaps may be considered [19]. Early skin flap repair is beneficial for the recovery/restoration of the local blood supply, which increases the likelihood of infection clearance. As the skin flap heals, it helps in joint movement, paving the way for future reconstructive surgery targeting joint function.

### Soft tissue management in two-stage revision

Joint stiffness is a significant problem in PJI [20]. The leading cause of joint stiffness is scar tissue formation resulting from infection of peri-articular soft tissues, especially in recently infected patients. Thus, resection of the scar tissue can facilitate joint exposure. Moreover, surgical removal of infected scar tissues surrounding the joint may help increase postoperative ROM, or even achieve normal ROM. For two-stage revision, an articular spacer could improve ROM one-year post-surgery [19]. One explanation for this may be that articulated spacers enhance soft tissue compliance during ROM, and the soft tissue around the joint can be further released during the second operation to improve ROM. Although the reported data varied substantially, existing literature showed that articular spacers did not lower infection control rates [21].

## Postoperative management of soft tissues

### General methods of managing postoperative wounds

Ensuring an adequate blood supply and avoiding wound-related complications are fundamentals in the prevention of wound problems [7]. Wound healing is a complicated process involving interactions among cells and mediators [22]. Pathophysiologically, robust blood flow is a critical basis for wound healing, which can be influenced by different wound closure techniques. In a randomized clinical trial, running subcuticular closure allows for the most physiologically robust blood flow, which may promote wound healing while staple closure resulted in the poorest blood flow [23]. Moreover, surgeons should strictly follow all necessary steps in postoperative care to ensure wound healing. Improving the patient’s nutritional status, proper use of vasodilators, and application of topical growth factors may facilitate the healing process [24]. Intermittent oxygen inhalation to increase the oxygen content in wound tissues can also help in wound healing [25].

### Postoperative wound dressing

Since the last century, researchers have discovered that wound healing is faster under moist conditions than in dry conditions when the wound is exposed to the air [26]. Moreover, wound oozing inhibits cell or tissue proliferation [27]. So far, a wide array of wound dressings are available. The ideal dressing for wound healing should be able to maintain a moist environment, absorb wound exudates, block bacterial invasion, and protect the wound surface from allergens [28].

Anti-septic ingredients in dressings may also aid in wound healing. Iodine and silver have been proven to be of anti-bacterial nature [28]. Iodine is effective against methicillin-resistant *Staphylococcus aureus* and reduces bioburden [29]. Researchers have also found that pathogenic organisms are eliminated at silver titers of 10–40 parts per million [30]. The chemical ingredients of dressings provide valuable options for surgeons to consider.

Negative pressure wound therapy benefits patients at a high risk of wound complications. Some possible reasons for this beneficial effect include reduced joint mobilization (thus stabilizing the wound’s boundary), elimination of dead space by exudate removal, improved blood flow, and a moist environment [31]. Therefore, negative pressure wound therapy should be considered to prevent wound complications in patients at a high risk of continuous drainage or with high tension at the incision.

### Rehabilitation

During the wound healing process, collagen type I, which increases/contributes to wound strength, accumulates approximately 3–4 days after surgery [32]. However, for patients with poor wound conditions and general conditions, the wound healing may be slower as compared to their counterparts with good conditions. If a patient starts rehabilitation of ROM too early, the risk of wound dehiscence is high, which may cause more problems, thereby impeding the whole rehabilitation process. Therefore, for patients with a high risk of wound complications, postoperative knee or hip joint exercise can be delayed for a period of time, e.g., two weeks.

### Dealing with the wound problem

Persistent wound drainage (PWD) after joint replacement is one of the challenges for orthopedic surgeons. For PJI revision patients, the surgeon must address the PWD concerns, whether it involves the first stage of spacer implantation or the second stage of joint reconstruction. Management of PWD consists of two steps: non-operative and operative protocols.

Non-operative protocols to deal with PWD include prophylaxically stopping venous thromboembolism, improving patient’s nutritional status, and using negative pressure wound therapy, as mentioned above.

If PWD lasts for more than 5–7 days postoperatively and the amount of drainage continues to increase, the patient must be subjected to re-operation. During debridement, a surgeon should evaluate whether the deep fascia is intact or not. If it is undamaged, the diagnosis of the wound condition is surgical site infection (SSI) rather than PJI and only a minimal debridement and suturing should suffice. If the wound has been carefully explored and the formation of the sinus has been verified, the diagnosis of PJI can be established. This would be akin to the Debridement, Antibiotics, and Implant Retention (DAIR) protocol. Given a virtually 60% overall infection control rate of DAIR and the advantages in cost-effectiveness, damage control, and rehabilitation [33], DAIR should be considered in all patients with acute PJI. All the modular components are recommended to be exchanged in the DAIR protocol. Any modifiable risk factors should also be addressed and corrected.

## Conclusions

Soft tissue management plays a critical role in treating periprosthetic joint infections. To improve the infection control rate and postoperative joint function, surgeons should be familiar with these general principles and rigorously apply them in clinical practice.

## Data Availability

Not applicable.

## Abbreviations

PJI: Periprosthetic joint infection
ROM: Range of motion
MRI: Magnetic resonance imaging
PWD: Persistent wound drainage
DAIR: Debridement, Antibiotics, and Implant Retention
